# Techniques for automated local activation time annotation and conduction velocity estimation in cardiac mapping

**DOI:** 10.1016/j.compbiomed.2015.04.027

**Published:** 2015-10-01

**Authors:** C.D. Cantwell, C.H. Roney, F.S. Ng, J.H. Siggers, S.J. Sherwin, N.S. Peters

**Affiliations:** a Department of Aeronautics, Imperial College London, South Kensington Campus, London, UK; b Department of Bioengineering, Imperial College London, South Kensington Campus, London, UK; c National Heart and Lung Institute, Imperial College London, South Kensington Campus, London, UK

**Keywords:** Conduction velocity, Cardiac electrophysiology, Local activation time, Cardiac mapping, Arrhythmias

## Abstract

Measurements of cardiac conduction velocity provide valuable functional and structural insight into the initiation and perpetuation of cardiac arrhythmias, in both a clinical and laboratory context. The interpretation of activation wavefronts and their propagation can identify mechanistic properties of a broad range of electrophysiological pathologies. However, the sparsity, distribution and uncertainty of recorded data make accurate conduction velocity calculation difficult. A wide range of mathematical approaches have been proposed for addressing this challenge, often targeted towards specific data modalities, species or recording environments. Many of these algorithms require identification of activation times from electrogram recordings which themselves may have complex morphology or low signal-to-noise ratio. This paper surveys algorithms designed for identifying local activation times and computing conduction direction and speed. Their suitability for use in different recording contexts and applications is assessed.

## 1 Introduction

Cardiac conduction velocity (CV) describes the speed and direction of propagation of the action potential wavefront through myocardium. It can provide important quantitative electrophysiological information about the underlying tissue microarchitecture and is widely used in both laboratory [1,2] and clinical electrophysiological studies [3,4] to infer properties of the myocardial substrate and to identify potential mechanisms for arrhythmogenesis [5–7]. Conduction velocity measurements provide an important quantity in identifying potential reentrant circuits and regions of tissue which, for example, might act as an anchor point for rotors [8]. Areas of tissue with slower conduction velocity are widely thought to be in a more diseased state, where either fibrosis or altered cell-to-cell coupling has reduced connectivity [9–22], or changes in the ionic currents such as the sodium current leads to slower action potential upstroke [23]. Slow conduction is associated with increased risk of wavefront reentry which may initiate an arrhythmia [24,25].

Besides the characteristics of the underlying tissue microarchitecture, other factors affecting propagation speeds include curvature of the wavefront [25–28], particularly around the infarct border zone [29–31]. A convex wavefront will propagate slower than a planar wavefront due to the depolarised region of tissue needing to excite a comparatively larger mass of cells. This phenomenon is known as source–sink mismatch (see, for example, [26,32]). In contrast, a concave wavefront advances with greater velocity due to a larger body of depolarised myocardium providing charge to a smaller region of non-excited tissue [31,33,34]. Structural branching of tissue is also known to impact conduction speed [35,36]. Velocity measurements can also be used to estimate anisotropic ratio [26] and they are found to be linearly related to the cardiac space constant [37].

The data modalities most frequently encountered in the context of conduction velocity estimation are the extracellular electrogram [9,12,13,38,39] and optical recordings of myocardium [40–42] or cell cultures using voltage-sensitive fluorescent dyes, in which changes in the optical signal are proportional to those in the transmembrane voltage. Calculation of the CV of an activation wavefront requires knowledge of both the speed and the direction of activation – the angle between the normal to the wavefront and the axis joining the measuring points. Propagation speeds are empirically determined based on relative distances and differences in times of local tissue activation, in the direction perpendicular to the wavefront. Directional information cannot be inferred from two recordings alone. Computing CV therefore requires at least three noncollinear measurement points, but frequently a larger number are used to minimise the impact of uncertainty in the acquired data. This is made particularly challenging when the underlying myocardium contains significant spatial heterogeneity of CV.

Distances between measurement sites are usually known, within a reasonable tolerance. For example, laboratory electrode arrays have a predefined precision arrangement, optical mapping has a calculable pixel diameter and the spacings between electrodes on a non-deformed multi-pole catheter are also known. In contrast, the annotation of local activation times (LATs), on often complex and noisy fractionated electrogram signals or optical mapping recordings, can sometimes be difficult to define. This has led to the use of other approaches to analyse these signals [43], including phase mapping [44,45], and frequency domain analysis [46,47]. However, overcoming the challenge is essential for accurate CV estimation.

In the laboratory environment, data are frequently collected using regularly spaced micro-electrode arrays [6,38] or optical mapping, both of which can provide high-density recordings over areas ranging from a few cells to entire hearts. In a clinical environment, the data modality is typically that of the unipolar or bipolar extracellular electrogram [48] and noise and far-field effects often complicate analyses. Spatial resolution is typically coarse, in comparison to some of the characteristic scales of the underlying tissue excitation. Electrogram data are often recorded independently or in combination with spatial location using an electroanatomic mapping system [49–51].

Activation maps may be constructed using concurrent recordings (e.g. using electrode-array catheters [52]) or, for stable rhythms, through sequential mapping. Multiple spatially distributed recordings obtained using a single- or multi-electrode catheters are recorded sequentially and the activity is synchronised based on the activation at a fixed reference point [53–55]. Non-contact catheters may also be used [25,52,53,56–58]. Approaches have been devised using specialised catheters specifically to estimate wavefront direction and speed in laboratory settings. For example, a catheter consisting of three electrodes arranged in an equilateral triangle around a fourth reference electrode allows the estimation of the direction of propagation and conduction speed based on the differences in measured activation times [59]. Although the approach was successfully tested in vivo in animal studies, it is not used clinically. It should be noted from the outset that conduction velocity in intact myocardium is a three-dimensional phenomenon. This is discussed further in Section 4.2.

The current generation of clinical electroanatomic mapping systems does not support the real-time construction of conduction velocity maps, necessitating off-line custom analysis [60]. Calculations of conduction speeds during procedures are therefore often manual and approximate [61], owing to time considerations. The inclusion of accurate automated localised conduction velocity estimation within electroanatomic mapping systems would enable clinicians access to this valuable metric during catheter ablation cases. Post-procedural investigations allow more precise computations, although approaches frequently remain manual and slow [61–63].

Finally, the potential of activation times and conduction velocity as metrics to elicit structural properties of myocardium is also of importance in developing increasingly accurate personalised computer models of electrophysiology or, alternatively, for their validation [64]. These models might be used as clinical diagnostic tools or to assist in the development and testing of proposed treatment [65]. Conduction velocity measurements can corroborate structural information discerned from non-invasive magnetic resonance imaging, as well as being directly integrated into the model [66].

There is a large body of literature on activation time and conduction velocity estimation and mapping. Earlier reviews within the scope of this literature have focused specifically on the analysis of high-resolution mapping data [67], directionality methods [68] and, more recently, optical mapping techniques [40]. The most appropriate technique to calculate CV depends on the type of recording (action potential or electrogram), spatial distribution of recording points (resolution and area of coverage), as well as the number of underlying wavefronts and their curvature. This paper therefore reviews currently available LAT and CV algorithms, assesses the applicability of each technique for various recording modalities, and recommends the most suitable technique for various datasets.

## 2 Local activation time

**Fig. 1:**
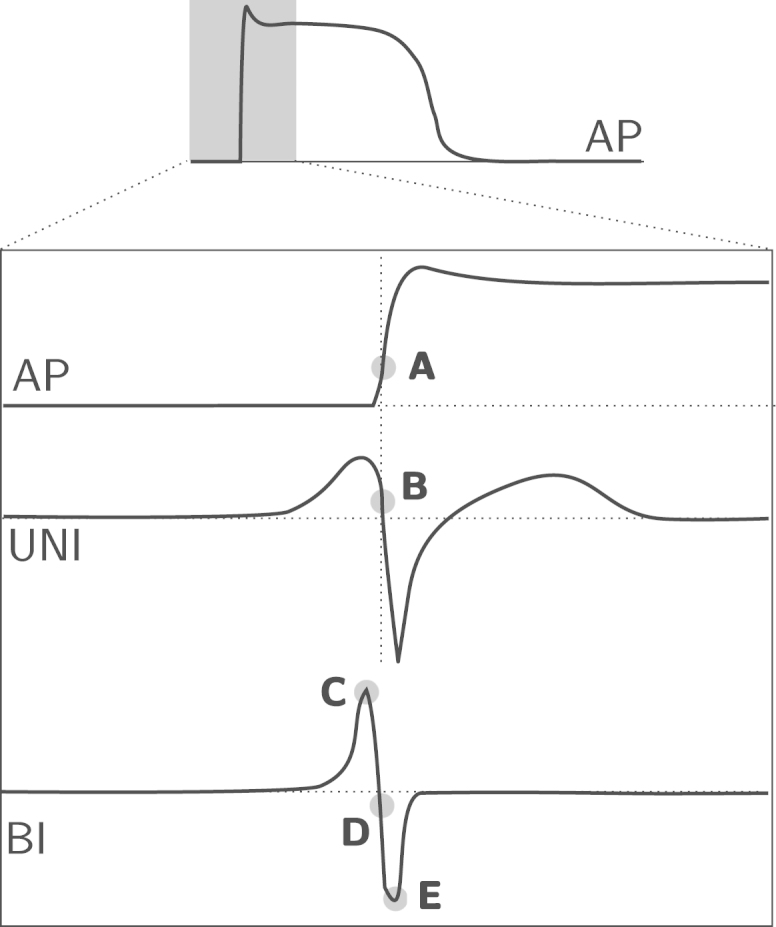
Diagram showing the location of commonly used activation times in the literature for the action potential (AP), extracellular unipolar (UNI) and bipolar (BI) electrograms. (A) Maximum dV/dt, (B) maximum negative dV/dt, (C) maximum absolute voltage |V|, (D) maximum absolute slope |dV/dt| and (E) minimum voltage.

The two most common clinical data modalities obtained from direct electrical observation of cardiac tissue are the unipolar and bipolar extracellular electrograms, measured by electrodes placed near or in direct contact with cardiac tissue [69]. Electrograms record the potential difference between two points and represent a summation of surrounding cellular electrical activity, the action potential, within proximity of the electrode locations. In a laboratory setting, processing and analysis of cardiac optical mapping data, obtained using potentiometric dyes can be used to visualise the action potential in ex vivo situations [40,41]. Analysis of cardiac conduction typically requires the identification of a tissue activation time in all of these signals. The time of maximum change in cellular transmembrane voltage is a widely accepted definition of the time of activation in the action potential (see Fig. 1). This maximal change in voltage has been quantitatively correlated with the peak conductance of the sodium channel [70], which initiates the depolarisation process in cardiac cells.

### 2.1 Unipolar vs. bipolar

Unipolar electrograms record the difference in electrical potential between an exploratory electrode in the heart and a fixed reference electrode a significant distance away. In contrast, bipolar electrograms are recorded between two electrodes of relatively close proximity within the heart. Bipolar electrograms contain only local electrical activity since they are recorded using differential amplifiers, leading to the rejection of far-field signals [48].

The maximum downslope of the unipolar electrogram is now considered the most accurate marker of local tissue activation [69]. A relationship has been demonstrated between extracellular recordings and the action potential, with the time of maximum $dV_{m}/dt$ in the action potential corresponding to the time of maximum $-dV_{e}/dt$ in the extracellular waveform [71], as shown in Fig. 1. There is, in fact, a quantitative relationship between both of these and the time of maximum sodium conductance, which all provide a marker for the same point in the depolarisation process [72].

The presence of far-field information in unipolar electrograms makes accurate identification of activation times in clinical electrograms challenging, leading to the routine use of bipolar electrograms during clinical practice [48]. However, bipolar electrograms are sensitive to inter-electrode spacing, wavefront orientation with respect to the inter-electrode axis [73], and the exact spatial location of the measurement is not clear. The ability to determine activation times from unipolar and bipolar electrograms has been compared in the clinic, laboratory [74] and through computer simulation [75].

In contrast to unipolar electrograms, the choice of marker of activation in the bipolar signal varies among the literature [69], with some of the common choices illustrated in Fig. 1. All three activation-time definitions are likely to produce accurate activation times with high-quality bipolar signals. However, choosing the minimum or maximum of a bipolar complex will most likely be more robust in fractionated signals, or low amplitude signals where the incident wavefront is almost parallel to the bipolar axis. Since the exact location of the measurement is unclear, unipolar activation times are preferred when a precise or absolute local activation time is required for comparison with other quantities [69].

To overcome the challenges of local activation time annotation, a number of other algorithms have been proposed. These are detailed in the remainder of this section.

### 2.2 Morphological approaches

**Fig. 2:**
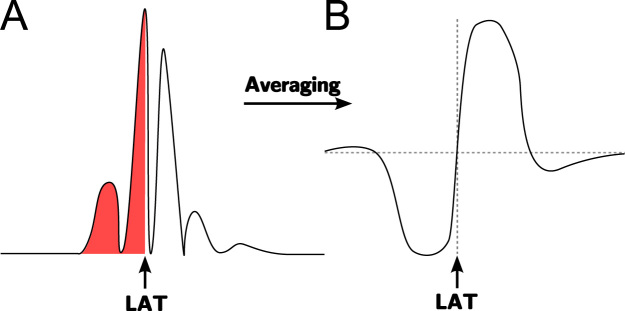
Diagram of morphological approaches. (A) Activation time defined as the point in the complex which equally divides the area under the modulus of the signal. (B) Using an averaging filter on the absolute value of the electrogram to identify the barycentre as the positive zero-crossing point as indicated.

For signals with high complexity, a morphological approach can be used. It is potentially less ambiguous than conventional bipolar electrogram markers since it is not dependent upon a single data point in the signal. In this method, as illustrated in Fig. 2, the point in the electrogram complex is chosen which equally divides into two the area under the modulus of the signal [76]. This method was found to be more accurate than the traditional maximum peak and maximum slope, based on expert manual estimation. The term *centre of mass* has also been used to describe this approach and has been found to coincide with the maximum slope in the unipolar electrogram [77]. While the above method identifies the fiducial point using the positive zero-crossing of a non-causal filtered signal (Fig. 2b), another approach is to simply fit the unfiltered signal to a cubic spline and find the point which equally partitions the enclosed area [78]. Morphological approaches have also been found to outperform the traditional bipolar markers when compared directly against unipolar activation times [74,79].

### 2.3 Non-linear energy

The non-linear energy operator (NLEO) is a measure of the energy of a signal and is proportional to the square of the product of signal amplitude and frequency [80]. For a single-component time-series of samples *x*_*j*_, this quantity can be expressed as

$$E_{j}=x_{j}^{2}-x_{j+1}x_{j-1}.$$

The NLEO can be used to identify active and inactive regions of the signal and subsequently calculate the proportions of each for use as a measure of electrogram fractionation [81]. Alternatively, the NLEO provides a technique for identifying activation times , and may better represent the true activation of tissue at the point between the bipolar electrodes than the conventional measures [82,83].

### 2.4 Time-delay cross-correlation

The traditional maximum gradient and signal peak markers for activation in electrogram signals may be difficult to identify or be unreliable if the sample rate of the data is too low or the morphology of the deflection is fractionated, such as for electrograms obtained from diseased tissue. For spatially local electrodes, activation time-delays between nearby recordings may be reliably measured, instead of through differences between absolute timings, by using a cross-correlation of the filtered signal [84]. This approach leads to a smaller standard deviation than that of maximum negative slope and therefore leads to more precise and reproducible time delay measurements [84]. The technique should only be considered robust when the signal morphologies are sufficiently similar since it makes the assumption that electrograms on different electrodes are related by a temporal shift. The method has been successfully used for experimental recordings with interelectrode spacings of just 0.69 mm in which activation times differed by <1ms [85].

Time delays have also been used for constructing *global activation maps* by measuring the activation delays between neighbouring electrodes and then choosing absolute times for all electrodes which best fit the delays [86]. To find the activation times $T={\left[T_{1},\ldots ,T_{N}\right]}^{⊤}$ at the *N* electrodes, a matrix problem is solved in the least-squares sense,

$$T={\left({\mathrm{DD}}^{⊤}\right)}^{-1}\mathrm{Dt}+T_{N},$$

where t is the vector of time differences *τ*_*ij*_, and D captures the relationship between them, $T_{i}-T_{j}=\tau_{\mathrm{ij}}$. *T*_*N*_ is set to $\min_{i}\;T_{i}$, such that the activation time of the earliest electrode is zero, making the problem well-defined. The method has also been extended to compute *directional activation maps*, see Section 3.11, and a similar approach has also been applied to optical signals [87].

### 2.5 Wavelet decomposition

Wavelet decomposition approaches to identifying electrogram activation times have been explored for ventricular electrograms [88]. Through careful selection of the prototype wavelet, this method identifies *maximum modulus lines*, defined as maxima and minima across the different scales of the transform, and through the relationship between the wavelet transform and the derivative of the signal, enables identification of the onset of activation.

The wavelet transform has also been used with optical mapping data [89] to remove motion artifact from optical action potential recordings. The decomposition of the signal and reconstruction from different scales allows the separation of noise, the early phase of the action potential and the motion.

### 2.6 Deconvolution

Convolution is a process frequently used to filter signals. The generation of an electrogram recording itself can be framed as a convolution of transmembrane potentials. The process of deconvolution can therefore be used to extract localised tissue activation [90]. The convolution operator is derived from the volume conductor equation, while a constrained minimisation algorithm is used to identify parameters of the forward model and minimise the difference between it and the observed electrogram. Although this approach assumes that the tissue is activated by a constant-velocity uniform wavefront, comparison with standard metrics and expert opinions, using simulated electrograms from which exact activation time is known, showed that the deconvolution approach is accurate, even for varying degrees of fractionation.

### 2.7 Template matching and libraries

Template matching is an automated process of comparing segments of an electrogram signal, or specific electrogram complexes, to a library of deflection morphologies. The library of reference complexes may be generated mathematically [91,92], or directly from actual electrogram recordings [93]. The approach is primarily targeted at identifying activation times during fibrillatory activity where multi-deflection complexes, whose morphologies vary over time, are present. Input signals are compared with the library recordings through a correlation function, in which maxima are sought and indicate a strong similarity of the template to the signal segment. These approaches have been applied with some success for signals recorded during atrial fibrillation, but can struggle to correctly annotate multi-component signals. In addition to the correlation function, the use of an error estimator may improve the robustness of the activation detection [93].

### 2.8 Multi-signal spatial methods

The use of spatial voltage gradients and the surface Laplacian between multiple electrodes have both been shown to produce an improved measure of activation time than standard time-derivative approaches, particularly for fractionated electrograms [94]. For spatial gradients, the maximum gradient is used as the activation time, while for the surface Laplacian the zero crossing closest to the maximum derivative is used.

### 2.9 Wavefront-tracking methods

Although not strictly a method for identifying local activation time, this approach is used to identify and track distinct activation wavefronts in data gathered from electrode arrays [95,96]. An electrode is considered *active* when the first derivative in time (dV/dt) is below a threshold *t*_*a*_. Wavefronts are constructed by locating active electrodes and flood-filling those surrounding pixels in the immediate neighbourhood which are also active. Subsequent samples in time are examined similarly, using previously established wavefronts as seeds, but also seeking any new wavefronts. Poor signals are replaced by an average of surrounding signals, rather than extending the neighbourhood to minimise the risk of artificially combining wavefronts. The collision and fragmentation of wavefronts can be detected and directed graphs can be generated to represent this. The approach has also been applied to optical mapping data, where optical action potential phase is used to identify wavefronts [97].

## 3 Conduction velocity estimation

Conduction velocity is empirically defined as the distance travelled by a wavefront in a unit of time. At small scales with predominantly one-dimensional uniform propagation, a measurement of distance between two recording points and the time delay between them is often sufficient to provide an accurate estimate [13]. In a two-dimensional setting, one typically requires information at a minimum of three noncollinear electrodes within a plane to establish a velocity vector. Speed can be estimated if knowledge of wavefront direction is known a priori. However, a more careful consideration of how conduction velocity is estimated is required in some circumstances. This is particularly true when working at larger scales, with heterogeneous tissue and fractionated electrograms, especially in clinical environments where noise and uncertainty in electrode locations are higher.

In this section we provide an overview of methodologies developed for assessing propagation speed and direction, for different electrical and optical data modalities and recording environments.

### 3.1 Spatial resolution requirements

The resolution of the acquired data is important in determining the reliability of algorithms to estimate conduction velocity. This is particularly true for curved wavefronts or those with short wavelength features, as a higher resolution of data points is required to satisfy the spatial Nyquist criterion that the interelectrode distance must be less than half the smallest relevant spatial wavelength [1]. High resolution data is therefore particularly important when working with complex and heterogeneous activation wavefronts where the spatial scales of interest are small.

In selecting a suitable algorithm for computing wavefront propagation speed and direction, a balance must therefore be sought between the resolution of the computed vector field and the accuracy of the estimation. Highly localised estimations of velocity will be more susceptible to error due to the increased relative uncertainty of position and activation time measurements, while estimations on larger spatial scales will only provide an average velocity and therefore exhibit poor correlation with features of the underlying local substrate.

### 3.2 Triangulation

Triangulation techniques allow conduction velocity estimation from a set of arbitrary points on a surface, without imposing significant constraints on their spacing or distribution. The approach is therefore well-suited to the clinical environment, where collected data typically possess these properties, and potentially allows large numbers of vectors to be computed for the dataset to create a high-resolution vector field.

A catheter with a fixed equilateral triangle arrangement of unipolar electrodes and a reference electrode in the centre is probably one of the earliest examples of triangulation being used to compute conduction velocity in a clinical setting [4]. However, the method is generalisable to non-equilateral triangles. Selection of triplets of electrodes can be achieved manually, through selection by an operator, or automatically through techniques such as Delaunay triangulation [98] or edge completion [99]. Additional constraints are typically imposed during the selection of triangles to improve the quality of the estimated vectors and minimise the relative influence of measurement errors [62].

**Fig. 3:**
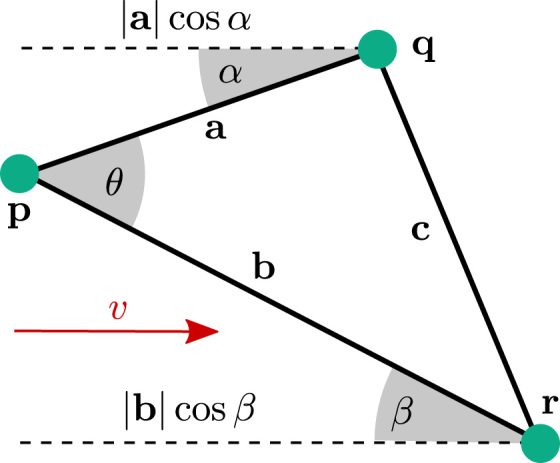
Diagram illustrating conduction velocity estimation through triangulation. *θ* is computed directly using the cosine rule from the known lengths a, b and c. The angle of incidence of the wavefront is calculated with respect to the sides a and b by angles *α* and *β*, respectively. These are determined through the time differences, distances and angle *θ*.

Using rules of trigonometry, the coordinates of three points can be used in association with their activation times to estimate the average conduction speed and direction within the enclosed triangle, assuming that the wavefront is approximated as locally planar. From the diagram in Fig. 3, for each triangle a relationship is derived between the speed and angle of incidence of the wavefront, as

$$\cos\beta =\cos(\theta -\alpha ),v=\frac{|a|\cos\alpha }{t_{a}},v=\frac{|b|\cos\beta }{t_{b}},$$

where *v* is the conduction speed, *θ* is the angle at the vertex **p** of earliest activation, computed as

$$\theta =\arccos(\frac{|a{|}^{2}+|b{|}^{2}-|c{|}^{2}}{2|a∥b|}).$$

The angles *α* and *β* describe the angle of incidence with respect to the two edges of the triangle meeting at **p**. Solving for *α* gives the direction of activation,

$$\tan\alpha =\frac{t_{b}|a|-t_{a}|b|\cos\theta }{t_{a}|b|\sin\theta },$$

and subsequently the speed *v* can be found.

**Fig. 4:**
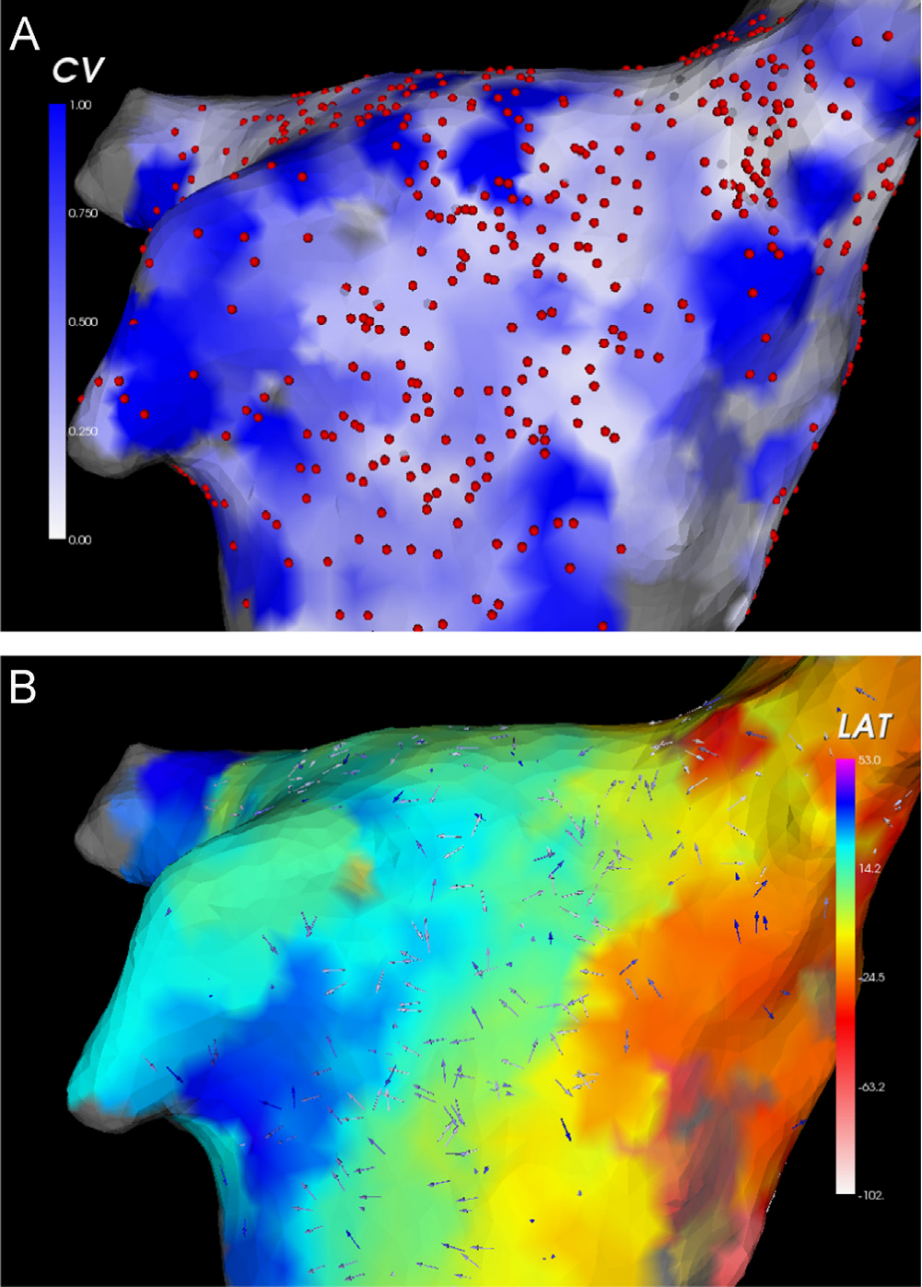
Example of conduction velocity maps calculated using triangulation of electroanatomic mapping data obtained during sinus rhythm. Data are interpolated up to a maximum distance of 5 mm. (A) Map of conduction speed. Regions of rapid conduction are shown in blue, while regions of slow conduction are shown in white. Circles denote locations of electrogram recordings. (B) Conduction velocity vectors, overlaid on a map of local activation. Earliest activation is shown in red, through to latest activation shown in blue. (For interpretation of the references to color in this figure caption, the reader is referred to the web version of this article.)

The approach has been used in a number of clinical studies [50,62,100,101], with global activation maps sequentially acquired during a stable rhythm. Constraints were imposed on distance (3≤d≤20mm), as well as the difference in activation times (>3ms), between vertices to reduce the impact of measurement errors. The method has also recently been automated [102] to generate high-density maps of conduction velocity from clinically acquired data. An example of this is illustrated in Fig. 4.

### 3.3 Finite difference techniques

**Fig. 5:**
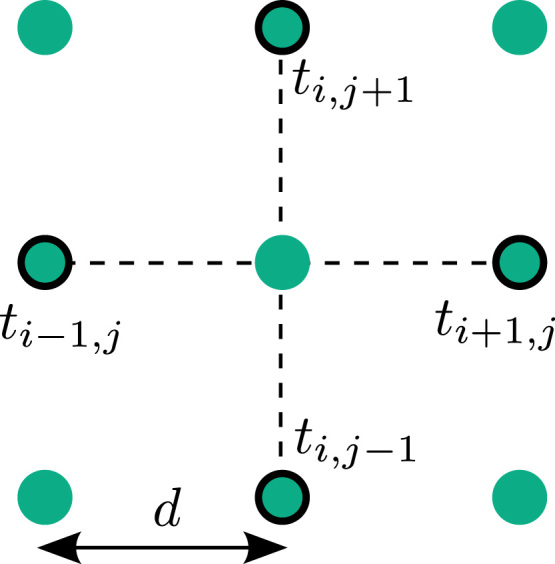
The finite difference technique uses measurements of activation time on an equally spaced grid with electrode separation *d*. Gradients of activation are computed along the dotted lines, in the horizontal and vertical directions, using the times at the four highlighted electrodes to calculate the conduction velocity vector for the centre point.

Finite difference methods are commonly used in the numerical solution of partial differential equations. Derivatives are approximated at a given grid-point, through differences between neighbouring grid-points, using a stencil as illustrated in Fig. 5. This approach can be used for computing local conduction velocity estimates at each point in the grid [42]. However, the technique requires that the data be located on a regularly spaced grid of points. It is therefore best suited for multi-electrode arrays or optical mapping data where the recording points are arranged in this manner.

The horizontal and vertical components of the gradient of activation are computed using standard first-order finite-difference stencils as

$$G_{x}=\frac{1}{2}[\frac{t_{i+1,j}-t_{i,j}}{d}+\frac{t_{i,j}-t_{i-1,j}}{d}]i=\frac{t_{i+1,j}-t_{i-1,j}}{2d}i,$$

and similarly

$$G_{y}=\frac{t_{i,j+1}-t_{i,j-1}}{2d}j.$$

The conduction speed |u| and the unit vector in the direction of activation, n^, are then given by

|u|=1|GA|=1Gx2+Gy2,n^=iGxGx2+Gy2+jGyGx2+Gy2,

leading to a velocity of

u=|u|n^=iGxGx2+Gy2+jGyGx2+Gy2.

**Fig. 6:**
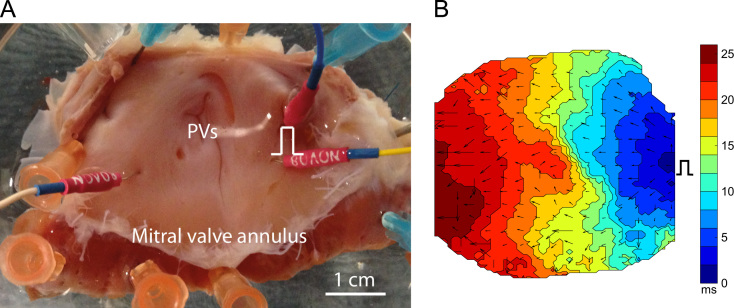
Example use of finite difference methods for computing localised conduction velocity from activation times derived from optical mapping data. (A) Photograph of canine left atrial preparation showing pacing electrodes and the location of the pulmonary veins. (B) Activation times recorded using optical mapping when the preparation is paced from the pacing point indicated. Conduction velocity vectors are computed using the finite difference method.

This technique has been applied in a number of optical mapping studies [40,103]. An example is shown in Fig. 6. The approach works well in situations where there is a high degree of tissue heterogeneity in the local conduction velocities. However, it fails when adjacent pixels have the same local activation time, such as when a low frame rate is used with optical mapping recordings [40].

### 3.4 Finite difference techniques with smoothing

**Fig. 7:**
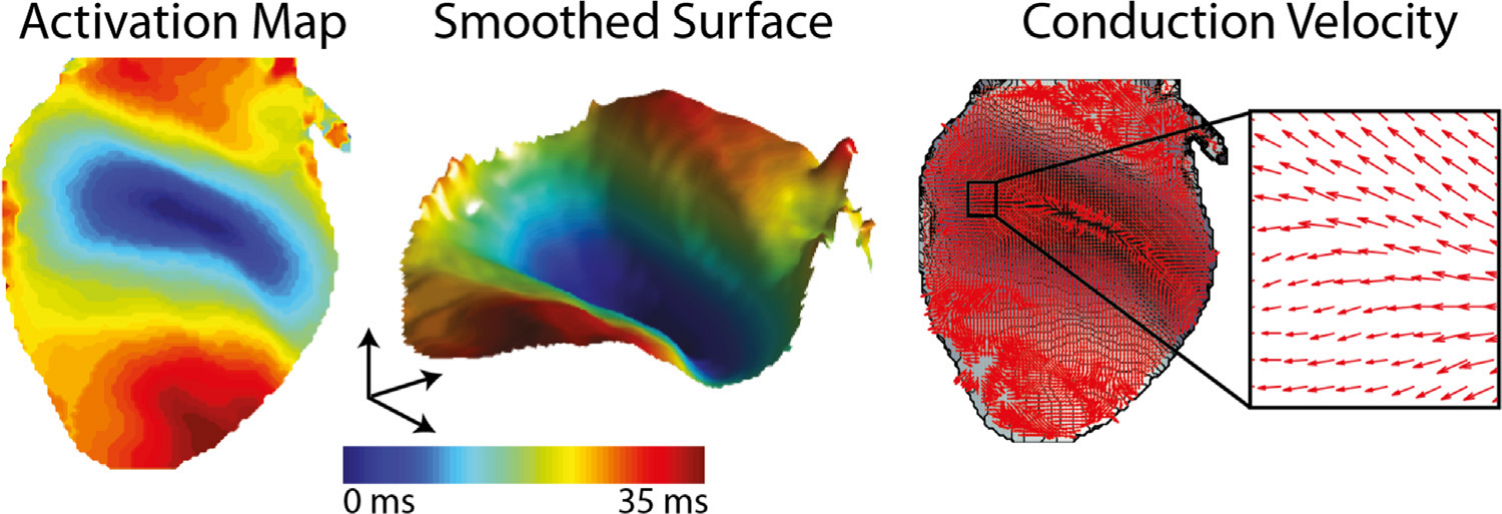
Conduction velocity map, generated using a smoothed finite difference approach, from optical mapping data. The smoothing is a 2D Gaussian convolution operator. Modified with permission from [40].

Finite difference approaches to computing conduction velocity are often susceptible to noise in the local activation time estimation or adjacent grid points having identical activation times, leading to spurious distortions of the conduction velocity field. This can be seen in the bottom left and centre of Fig. 6B where the arrows suggest unphysiological rapid localised variations in conduction direction. An approach to overcome this is through applying a convolution technique to *smooth* the local activation. An example of this is given in Fig. 7, where a two-dimensional Gaussian smoothing operator is used to reduce localised noise in activation times and produce a smoother conduction velocity vector field.

### 3.5 Polynomial surface fitting

This class of techniques fits one or more polynomial surfaces $T\left(x\right)=T_{k}\left(x\right)$ through subsets of the space-time coordinates $\left(\tilde{x},t\right)$, where $\tilde{x}$ is the electrode position, *t* is the wavefront activation time and *k* is the order of the polynomial surface. The surface is fit to the data using a standard least-squares algorithm. Although the method using quadratic surfaces has been applied to regularly spaced unipolar electrode arrays in the two-dimensional case [1], the arrangement of points may be arbitrary. This has been demonstrated in a later study using three-dimensional paced and sinus rhythm data [2], gathered using a plunge electrode. In these studies, activation of a given electrode was defined by dV/dt<−0.5V/s. At a given time, wavefronts were defined as being at locations where there had been no activity in the preceding 40 ms. The approach can also be applied to compute the propagation velocity of tracked wavefronts (see Section 2.9) [97].

To compute a conduction velocity vector at an arbitrary point $\overset{¯}{x}$ using quadratic polynomial surfaces, the data $\left({\tilde{x}}_{i},t_{i}\right)$ within a fixed neighbourhood of $\overset{¯}{x}$ is fit to the expression of the form

$$T(x,y)={\mathrm{ax}}^{2}+{\mathrm{by}}^{2}+\mathrm{cxy}+\mathrm{dx}+\mathrm{ey}+f.$$

The velocity vector is then defined as

$$v_{e}=(\begin{array}{c} \frac{dx}{dT}\\ \frac{dy}{dT} \end{array})=(\begin{array}{c} \frac{T_{x}}{T_{x}^{2}+T_{y}^{2}}\\ \frac{T_{y}}{T_{x}^{2}+T_{y}^{2}} \end{array}).$$

If there are more data points than parameters in the expression for the surface this acts to smooth the data and reduce the impact of outliers. Note that although the above leads to the same expression as for the finite difference technique (Section 3.3), the gradients in the case of polynomial surface-fitting techniques are evaluated analytically on the surface and therefore a vector may be computed at any arbitrary point for which there are sufficient data points within a neighbourhood.

To fit a quadratic surface, six data points are required, although twenty are typically needed for a good fit with two-dimensional data [1], or if the points are linearly dependent [2]. The neighbourhood size should therefore be several times the spatial sampling resolution. The least-squares fitting algorithm provides robustness against outliers. The use of a smooth surface also reduces the impact of noise through electrode position measurement or activation time determination. The residual of the least-squares algorithm provides a metric with which to assess the quality of the fit to the data. The method has been demonstrated to work well for simulated data, although some sinus rhythm and paced wavefronts were found to be too complex to capture in three dimensions with the available data [2].

The surface-fitting technique is frequently used with first-order surfaces (e.g. [104]), which leads to a method similar to standard finite difference approaches. A cubic polynomial surface variant has also been considered, which has twenty unknowns, and while it is found to provide a more accurate conduction velocity estimation for complex activation wavefronts it requires significantly more data points [105]. Quadratic and cubic surfaces were both found to underestimate curvature of the wavefront and the linear fit also led to incorrect speed estimations.

Other variants on the polynomial surface fitting method have been investigated. Surface fitting to activation time delays using small data sets has been considered [85]. Velocity vectors are estimated using four to seven electrodes, which makes the method potentially clinically applicable. The curvature of the heart surface is also important when applying the method to optical mapping data and, accounting for this, allows distances between data points to be more accurately captured, providing improved CV estimates [106]. Panoramic mapping techniques have also been developed to address curvature of the heart [107], although the technology is not widely available [40].

### 3.6 Cosine-fit techniques

**Fig. 8:**
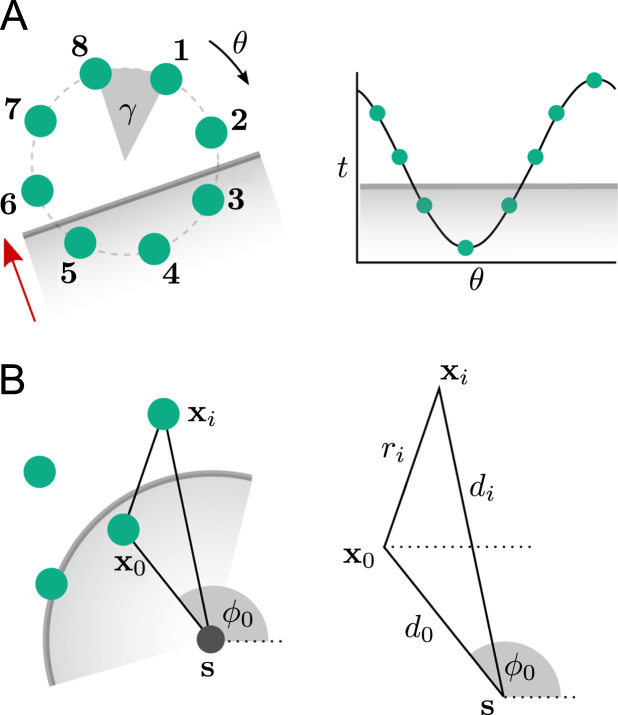
(A) Planar wave activation across a circular catheter, estimated using a cosine-fit technique. Activation times at the 8 electrodes are fit to the translated cosθ function as shown in a least-squares sense. (B) Circular wave conduction velocity and focal source, **s**, estimated from an arbitrary set of recording points at positions **x**_*i*_, with **x**_0_ being the point of earliest activation. The distances of each point from the focal source and **x**_0_ are denoted by *d*_*i*_ and *r*_*i*_, respectively.

In a clinical environment, measurement points are typically in a fixed arrangement depending on the choice of catheter and one is interested in the nature of the propagation of macroscopic wavefronts across the catheter to assist in diagnosis. For a planar wave passing over a circle of recording points with constant offset *γ* and radius *r*, illustrated in Fig. 8A, the activation times satisfy the following equation:

$$t(n)=t_{c}-A\;\cos[\gamma (n-1)-\phi_{0}],$$

where *t*_*c*_ is the centre activation time and *ϕ*_0_ is the angle of earliest activation [82]. Initial values of the unknowns *t*_*c*_, *A* and *ϕ*_0_ are estimated from the sequence of activation and a sequential quadratic programming algorithm is used to fit the parameters to the data. The conduction velocity is then estimated as r/A.

Initial validation of the method was provided through simulated data [82]. The detected direction of propagation was found to be moderately tolerant to Gaussian noise with standard deviation up to 20%, applied to the activation times and errors in inter-electrode angle *γ* of up to 3°. The influence of curvature of the incident wavefront was examined using two point stimuli 25 mm and 50 mm away from the nearest recording electrode. In this case the error in propagation direction increased by only 1.5°. However, the model is unable to correctly identify multiple concurrent wavefronts, and estimated angles for incident spiral wavefronts do not necessarily point directly towards the core.

This method has been applied to investigate human conduction velocity restitution properties using a circular multipolar catheter [108]. The method is robust to a small degree of curvature, so is appropriate when the pacing is a sufficiently large distance from the catheter. It has also been used to compare clinical circular catheter data collected during both sinus rhythm and paced rhythms to patient-specific simulated data [83].

Recently, the method has been extended to consider different catheter configurations and wavefront shapes [109]. The technique is generalised to support an arbitrary arrangement of points, thereby adapting better to clinically acquired data, and both planar and circular wavefronts. This enables prediction of the focal source location based on estimated wavefront curvature and is illustrated in Fig. 8B. A limitation of the approach is that the points are projected onto a two-dimensional plane of best fit, which will distort distances between electrodes and therefore introduce a slight error into the estimate of the focal source.

### 3.7 Vector loops and ensembles

The direction of activation can be inferred from the relative amplitude of two bipolar electrograms recorded from a custom electrode array consisting of two orthogonal pairs of electrodes [3]. The term *vector loop* originates from the use of an oscilloscope to process the bipolar electrograms through the X and Y inputs, resulting in a loop during activation. The direction of the signals departure from the origin indicates the direction of activation with respect to the bipole orientations.

Computing multiple activation vectors in this way at fixed locations provides a measure of the regularity of activation direction. In a later study, the vector loop method was used in conjunction with a 112-electrode array to investigate the consistency of propagation direction in AF [110]. Groups of four electrodes were used as a pair of orthogonal bipoles. The approach has also been extended to three dimensions in humans through a specially designed catheter and is found to reliably predict anterograde and retrograde conduction [111].

### 3.8 Radial basis function interpolation

Radial basis functions provide a technique for interpolating LATs across the endocardial surface, which allows activation patterns, including wavefront collision, to be detected. This class of functions, ϕ(x)=ϕ(∥x∥), are dependent only on the distance from a fixed point; an example is the Gaussian function $\phi (r)=e^{-{\left(\epsilon r\right)}^{2}}$. For activation times *t*_*i*_ corresponding to the *N* electrodes at positions $x_{i}$, an activation surface can be represented as a sum of radial basis functions,

$$T(x)=\overset{N}{\underset{i=1}{\sum }}\alpha_{i}\phi_{i}(∥x-x_{i}∥)+\overset{M}{\underset{j=1}{\sum }}\beta_{j}\psi_{j}(x),$$

where the radial functions *ϕ*_*i*_ in the first term are centred at the measurement points $x_{i}$, and the second term is the associated polynomial [112]. The constraints $T(x_{i})=t_{i}$ ensure that the surface matches the recorded activation times at the electrode positions. The linear system of *N* equations derived from the above expression is then solved to determine the coefficients *α*_*i*_. If the chosen radial basis function is not positive definite, additional low-order polynomials *ψ*_*j*_ and constraints may need to be added to ensure a unique solution of the interpolation problem [113].

Given the known activation surface, gradients of activation and subsequently conduction velocity can be calculated [112] in a similar manner to that used in Section 3.5. Activation times throughout a chamber can be determined from global activation maps and therefore high-density conduction velocity vector fields can be computed [114]. The ability to generate high-density vector fields also enables a number of other quantities such as divergence and curl to be investigated [112], which are briefly discussed in Section 4.4.

Wavefront collision and ectopic foci can also be detected through the use of radial basis function interpolation [115]. While the technique could detect foci with either a spiral or PentaRay catheter, it was not able to capture the source with the circular catheter, since this arrangement lacks radial information.

### 3.9 Isopotential lines

Conduction velocity can be estimated by considering the distance travelled by an isopotential line over a fixed time interval [116]. In this approach, at each time instant an isopotential line is constructed using a parametric spline fitted through those data points at a fixed potential. This can identify both the wave front and wave back, depending on the sign of dV/dt. The conduction velocity at a given point on the line is then estimated by examining the distance travelled in the direction normal to the isopotential line over a fixed time window. The normal vector is easily computed from the spline expression. This technique requires a higher resolution of data than is clinically available and necessitates absolute measurements of membrane potential, limiting its applicability to optical mapping.

### 3.10 Arbitrary scalar fields

A generalisation of the isopotential lines method (Section 3.9) is to use spatial gradients of any scalar quantity for which a specific isovalue corresponds to the excitation wavefront [117]. Examples of such scalar fields include activation time, electrical potential or electrical phase.

### 3.11 Time delays

Activation maps are potentially easier to compute in terms of differences between neighbouring electrodes, where electrogram morphology is expected to be very similar, rather than explicitly calculating the activation time of each electrogram independently (see Section 2.4). Extending this idea, time differences between electrodes in a small neighbourhood of electrodes can be used to estimate a plane-wave propagation velocity across the localised region [86].

For any given pair of electrodes in the neighbourhood, the wavefront velocity can be expressed as

$$v=\frac{(x_{j}-x_{i})\cdot n}{\tau_{\mathrm{ij}}},$$

where $x_{i}$ and $x_{j}$ are the locations of the electrodes, n is the unit normal to the planar wavefront and *τ*_*ij*_ is the time difference between them.

**Fig. 9:**
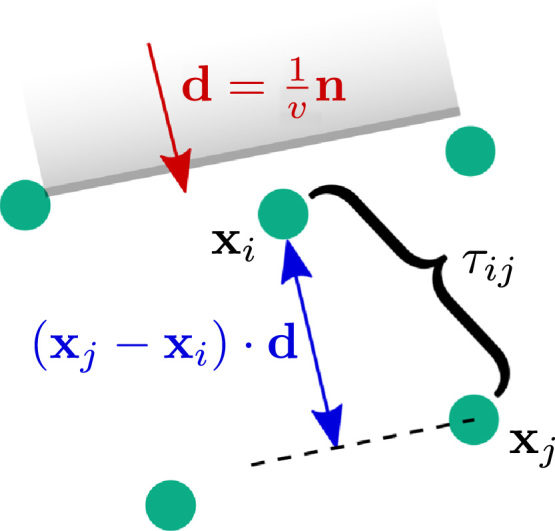
Estimation of planar wavefront velocity from differences in location and activation time. Expressions relating inter-electrode distances normal to the wavefront, $(x_{j}-x_{i})\cdot d$, and their corresponding time delay can be used to estimate **d**, and subsequently compute the wavefront speed, *v*.

Defining d=(1/v)n, a system of equations,

$$(x_{j}-x_{i})\cdot d=\tau_{\mathrm{ij}},$$

can be derived which relate inter-electrode distances in the direction of the wavefront with corresponding time delays, as shown in Fig. 9. This can be written in matrix form as

$$A^{⊤}d=t,$$

and solved in a least-squares sense, due to measurement error and the premise that the activation wavefront is not truly planar.

The approach has been validated on simulated data where a conduction velocity vector field was generated by using Delaunay triangulation [98] on the points and applying the method to each resulting triangle of electrodes. It is also worth noting the similarity between this method and the polynomial surface fitting with first-order surfaces (see Section 3.5). However, this method differs by the use of differences in space and time between electrodes to compute the velocity, rather than requiring explicit knowledge of the activation time at each electrode.

### 3.12 Analytic expressions

Expressions can be derived for wavefront curvature, speed and direction of propagation from a fixed stencil of 4 points surrounding a point of interest, provided that the spacing is significantly less than the radius of curvature of the wavefront [118]. The distances of an unknown focal source from each of the four points are expressed in terms of the distance to the point of interest and, given the activation times at each electrode, the equations are solved to compute wavefront velocity and radius of curvature. If one is only interested in conduction velocity, three points equispaced around a circle are sufficient.

The method makes the assumptions that propagation is smooth, continuous and normal to the wavefront and that the radius of curvature is large enough that it can be approximated locally as a circle. It also has a limitation that the radius is undefined when the angle of incidence is 45° and two pairs of electrodes are activated simultaneously. However, this can be overcome by adding a fifth measurement at the point of interest. The equations were tested on simulated data and verified against empirical estimates of conduction velocity.

### 3.13 Maximum likelihood estimation

**Fig. 10:**
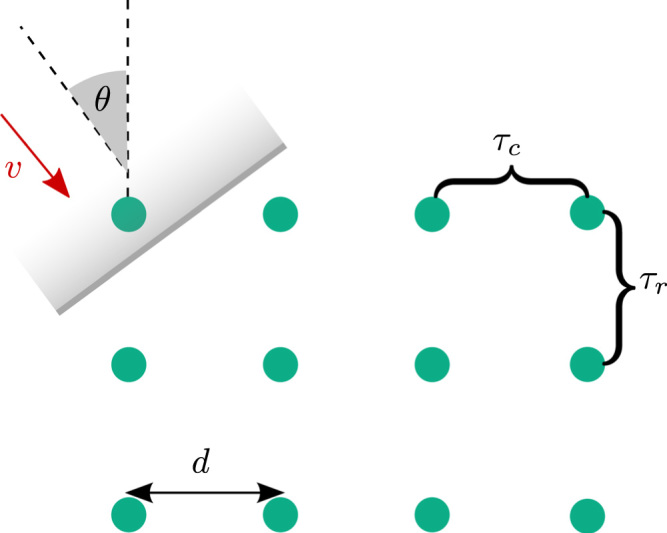
Planar wavefront velocity estimation from an equally spaced grid of electrodes using a maximum likelihood approach. The most likely row and column time delays, *τ*_*r*_ and *τ*_*c*_, are estimated from which the velocity can be computed using trigonometry.

Statistical approaches to measuring conduction velocity across high-density grids of electrodes have been considered for measuring fetal cardiac activity [119]. The wavelength of the signal is on the order of the size of the electrode grid and so the incident wave is assumed to be planar with incident angle *θ* and velocity *v*. In brief, the signals at each electrode are assumed to share the same morphology *s*(*n*) and therefore can be modelled as a time shift of this signal, based on the row *r* and column *c* of the electrode,

$$x_{\mathrm{rc}}(n)=s(n-(r-1)\tau_{r}-(c-1)\tau_{c})+\omega_{\mathrm{rc}}(n)$$

where ω(n) is Gaussian white noise with variance *σ*^2^ and *n* is the index of the sample. This is illustrated in Fig. 10. *τ*_*r*_ and *τ*_*c*_ describe the time delay between the rows and columns respectively and these are estimated by maximising the probability

$$p((\tau_{r},\tau_{c})|x_{\mathrm{rc}}(n),s(n)).$$

Through the use of Bayesian inference, the maximum likelihood estimation of $\left(\tau_{r},\tau_{c}\right)$ can be reduced to the minimisation of a cost function. The maximum likelihood estimation method uses weights in the cost function which depend on the signal-to-noise ratio and this is found to significantly improve the accuracy of the estimation. Subsequently, the conduction speed and angle of incidence can be computed through the expressions

$$v=\frac{f_{s}d}{\sqrt{\tau_{r}^{2}+\tau_{c}^{2}}},$$

and

$$\theta =\cos^{-1}(\frac{\tau_{r}}{\sqrt{\tau_{r}^{2}+\tau_{c}^{2}}}),$$

respectively, where *f*_*s*_ is the sample rate and *d* the inter-electrode distance.

## 4 Discussion

### 4.1 Comparisons of conduction velocity algorithms

We list in Table 1 the conduction velocity estimation techniques reviewed in this paper and their applicability to different data modalities and resolution constraints. For a clinical environment the most suitable techniques are triangulation, cosine-fit algorithms and radial basis functions. For localised single-catheter analysis the cosine-fit technique is robust. Triangulation has the greatest potential for use in rapid conduction velocity mapping as it can be applied globally and is computationally less expensive than using radial basis functions. However, for very high-density maps, triangulation may be overly sensitive to measurement error due to the small size of triangle used. Radial basis functions my be more resilient in this case and allow a high-density vector field to be generated, independent of the set of recording points, which also supports the use of vector field analysis.

Both finite difference and polynomial surface fitting techniques are widely used in the literature with optical mapping recordings and micro-electrode array data. Although finite difference approaches are straightforward to implement, they are not as effective at handling missing data as polynomial surface-fitting techniques [1]. For regions of heterogeneous CV, which cannot be easily described by polynomials, smoothed finite difference approaches are found to be superior [40]. Paskaranandavadivel et al. [120] also compared regular finite difference methods, smoothed finite difference methods and the polynomial-fitting techniques in the context of gastric slow-wave propagation, concluding that smoothed finite difference gave the most accurate results. Techniques involving computing iso-scalar lines are also suitable for use with high-resolution optical mapping data, although their implementation is more complex than the previous methods and may therefore make them less desirable.

### 4.2 Three-dimensionality

Propagation wavefronts are three-dimensional in intact myocardium. Many of the techniques outlined in this review operate on two-dimensional data collected from either the epicardial or endocardial surface and are therefore inherently limited in their ability to determine true wavefront speed. The conduction velocity of wavefronts that are not travelling exactly tangential to the recording surface will be over-estimated. This is especially true in thicker structures, such as the ventricular walls, where transmural propagation is common. However, it should be noted that this is a limitation of the recording technology and many methods could work with volumetric data equally well. One example is the polynomial surface fitting technique which has been extended to compute three-dimensional wavefronts using data recorded using plunge electrodes in a volume of tissue [2].

### 4.3 Relationship with other quantities

The relationship between CV and other functional and structural factors has been investigated in many clinical studies, with the motivation of understanding the electroanatomic substrate underlying cardiac arrhythmias in order to guide ablation therapy. CV has been found to correlate with bipolar electrogram amplitude in atrial flutter reentry circuits [121], where a logarithmic relationship was found. This could be used to directly predict local CV from measured electrograms. A correlation was also found during sinus rhythm, for patients who had a history of AF, between the areas of lowest bipolar electrogram voltage (<0.5 mV) and low CV, which often colocalised with fractionation and double potentials [122]. However, changes in propagation velocity are not always associated with changes in electrogram duration [123]. Electrogram fractionation may indicate conduction slowing [124] and fractionation of sinus rhythm electrograms has been shown to correlate with age, voltage and CV [125]. Peak negative voltage of unipolar electrograms has been shown to correlate with conduction slowing in patients with atypical right atrial flutter [126].

The rate dependence of CV has been shown to be a more important indicator of AF initiation than electrogram fractionation, where conduction was seen to slow immediately prior to AF [127]. Although CV restitution is not routinely measured in clinical cases, CV restitution has been characterised in humans using cosine-fit techniques (see Section 3.6) with a circular catheter [82].

Late-gadolinium enhanced magnetic resonance imaging (LGE-MRI) has been used to identify areas of fibrosis and delineate scar tissue in patients with AF [128,129]. The correlation between LGE-MRI image intensity and CV is an area of active research [130,131].

### 4.4 Secondary analysis of velocity vector fields

Local normalised CV vector fields can be further analysed by applying vector calculus operations to elicit a more qualitative interpretation of the data. The divergence of the two-dimensional CV vector field,

$$\nabla \cdot v=\frac{\partial v_{x}}{\partial x}+\frac{\partial v_{y}}{\partial y},$$

can be used to distinguish between focal sources and areas of collision. Normalisation ensures only the direction of the vectors influences the divergence. At a source, all of the conduction velocity vectors point outwards resulting in a positive divergence; at a sink or area of collision, the divergence will be negative. The curl of a two-dimensional CV vector field,

$$\nabla \times v=(\frac{\partial v_{y}}{\partial x}-\frac{\partial v_{x}}{\partial y}),$$

computes twice the local angular velocity, with positive curl indicating counterclockwise rotation and negative curl indicating clockwise rotation.

The divergence and curl operators have been applied to CV vector fields from simulated [132,112], canine epicardial electrograms [133] and human atrial LAT maps [133]. These operators require a regular grid of CV vectors, which can be obtained from irregularly arranged data in several ways. Radial basis function interpolation (Section 3.8) can be applied to the activation times, followed by finite difference methods [115] (Section 3.3) or polynomial surface fitting methods [112] (Section 3.5) to calculate the CV vectors. Fitzgerald et al. [133] calculated the divergence of human atrial LAT data from the electroanatomic system Carto by fitting the electrogram positions to an ellipsoid, projecting onto a 2D plane, spatially interpolating the LATs and finally using a linear polynomial fit to the data [85]. In order to accurately locate ectopic foci, spatial resolution can be improved by Delaunay triangulation and cubic interpolation [133]. In addition, the use of the Radon transform has been suggested to allow more accurate localisation of areas of high divergence [115].

Ectopic foci have been successfully identified using divergence maxima, providing the CV vectors surround the foci [112,115,133]. Uniform spacing is not required and this technique has been applied to simulated data and high-density circular, spiral and five-spline mapping catheters [112,115]. Localisation is accurate for a five-spline catheter when up to eight of the fifteen recording points were missing as a random distribution although the removal of two entire splines of data may change the ability of the vector field analysis to identify complex activation patterns. Divergence is low in areas of wavefront collision, where collision was confirmed by the presence of double potentials for human clinical data [133]. However, using the same dataset, curl did not indicate any central obstacles in reentrant circuits.

### 4.5 Open questions

Activation time mapping and conduction velocity mapping are important metrics for understanding the structural and functional electrophysiology in both the laboratory and clinical environment. However, challenges still remain:

• Identifying local activations in complex fractionated signals consisting of many low-amplitude deflections. A greater understanding of the electrogram and its decomposition in terms of local cellular activity is needed.
• Conduction velocity mapping during atrial fibrillation would improve the identification of focal sources and those regions of the chamber perpetuating arrhythmogenic activity.
• Real-time generation of complete-chamber conduction velocity mapping during simple rhythms is needed to augment existing clinical diagnosis of arrhythmias.
• Estimating the level of uncertainty in computing the conduction velocity of propagating wavefronts in three-dimensional tissue using two-dimensional surface measurements and algorithms.

## Conflict of interest statement

None declared.

## Figures and Tables

**Table 1:** List of conduction velocity techniques and their advantages and disadvantages. Suitability of the methods to different data modalities and any restrictions on the type of data are also noted.

Method	Advantages	Disadvantages	*Suitability* and requirements
Triangulation [4,62,100–102]	Local score, examine regional heterogeneities, any arrangement of points, uses actual LATs	Sensitive to error in LAT, difficult to automate	*Clinical,*3≥d≥20mm, LAT differences >3 ms
Finite difference [42,40]	Local score, examine regional heterogeneities, easy to implement, uses actual LATs	Sensitive to noise / missing data, Fails if times are identical, Requires regular grid	*Optical mapping, Multielectrode arrays,* 4 points, sufficient temporal resolution to avoid adjacent equal activation times
Polynomial surface [1,2,85,106,40]	Any arrangement of points, robust to noise, allows missing data points, residual to assess quality of fit	May require more points than available, requires choice of ΔX,ΔT	*Optical mapping, Multielectrode arrays,* 3D: linear (4 points), quadratic (10 points), cubic (20 points), more points needed for complicated rhythms
Cosine-fit [82,108,83,109]	Measure of curvature and distance to focal source, any arrangement of points, robust to noise, residual to assess quality of fit	Single macroscopic wavefront only, one vector per catheter	*Clinical,* no colliding wavefronts
Vector loops [3,110,111]	Does not require LAT assignment	Requires specific catheter	*Clinical,* 2 orthogonal pairs of bipoles
Radial basis [112,114]	Multiple wavefronts, use to find LATs anywhere on surface, no assumption on arrangement and spacing, high res. velocity field (div, curl)	Computationally demanding	*Clinical,* any arrangement
Isopotential lines [116]	Accurate wavefront curvature estimation, robust to spatial noise	Requires measurements of membrane potential, requires high resolution, LATs do not always coincide with isopotential lines	*Optical mapping,* high resolution
Arbitrary scalar fields [117]	Extends CV calculation from isopotential lines to use other variables	Requires measurement of another scalar field	Scalar field (e.g. activation time, electrical potential, phase)
Time delays [86]	Uses neighbouring location information, can deal with incorrect LATs, local score, Any arrangement of points	Assumption of plane wave locally	*Clinical*
Analytic expressions [118]	Velocity and curvature from 4/5 points, low density data, simple to apply	Points must lie on a square, radius of curvature must be large, requires accurate LATs	*Optical mapping*, *multielectrode arrays*, points on a square
Maximum likelihood [119]	Statistical approach, tolerant of LAT measurement errors	Requires grid of recording points	*Multielectrode arrays,* equally spaced grid of points
